# Expressions and clinical significance of serum bone Gla-protein, bone alkaline phosphatase and C-terminal telopeptide of type I collagen in bone metabolism of patients with osteoporosis

**DOI:** 10.12669/pjms.311.6143

**Published:** 2015

**Authors:** Dongfeng Zhao, Junsheng Wang, Yining Liu, Xinwei Liu

**Affiliations:** 1 Dongfeng Zhao, Department of Orthopedics, The 210th Hospital of People's Liberation Army, Dalian 116021, PR China.; 2 Junsheng Wang, Department of Orthopedics, The 210th Hospital of People's Liberation Army, Dalian 116021, PR China.; 3 Yining Liu, Department of Orthopedics, The 210th Hospital of People's Liberation Army, Dalian 116021, PR China.; 4 Xinwei Liu, Department of Orthopedics, The 210th Hospital of People's Liberation Army, Dalian 116021, PR China.

**Keywords:** Osteoporosis, Bone Gla-protein, Bone alkaline phosphatase, C-terminal telopeptide of type I collagen

## Abstract

**Objective:**

This study aimed to evaluate the expressions and clinical significance of bone Gla-protein (BGP), bone alkaline phosphatase (B-ALP) and C-terminal telopeptide of type I collagen (CTX) in patients with osteoporosis (OP), and to provide evidence for developing individualized treatment plans.

**Methods:**

Seventy-two OP patients in our hospital were selected as an OP group, and another 72 healthy subjects were used as a control group. Their BGP, B-ALP and CTX levels as well as bone mineral density (BMD) values were measured. The correlations between BGP, B-ALP and CTX levels and BMD values were determined.

**Results:**

The BGP level of the OP group [(5.61±5.52) ng/ml] was significantly higher than that of the control group (P<0.05), but the levels of B-ALP and CTX did not differ significantly (P>0.05). The BMD values of femoral neck and Ward's triangle in the OP group were negatively correlated with the B-ALP levels (P<0.05). For women OP patients, the BMD values of femoral neck and Ward's triangle were also negatively correlated with the B-ALP levels (P<0.05). The BMD of femoral neck in the control group was negatively correlated with the CTX level (P<0.05).

**Conclusion:**

Determining BGP, B-ALP and CTX levels can evaluate the bone metabolism degree, which provides evidence for clinical typing of OP and developing treatment strategies.

## INTRODUCTION

Osteoporosis (OP), a systemic skeletal disease characterized by low bone mineral density (BMD) and microarchitectural deterioration of bone tissue, induces bone fragility and increases fracture risk.^1^ Especially, it is prevalent in elderly postmenopausal women.^2^ Osteoporotic fracture is prone to development partly because prior fracture is an important independent risk factor.^3^ Moreover, women with extremely low BMD have much higher risks than the average. As the most serious consequence of osteoporosis, hip fracture elevates the odds of women deaths by 2-4 times in the general population, but there remains controversy over the relationship between deaths and comorbid conditions or hip fracture *per se*. Even after surviving 12 months posterior to a hip fracture, patients are subject to severe functional declines. It has previously been reported that 50%, 70%, and 87% of the women with hip fractures failed to independently walk, transfer from one place to another, and climb stairs respectively.^4^

With unraveled pathogenic mechanism, OP has been associated with the level of calcium intake, genetic factors, bad habits and hormone secretion, etc.^5^ Particularly, the patients in critical conditions may suffer from intense pain and frequent spontaneous fractures, with significantly affected health and quality of life.^6^ Generally, OP should be treated according to the status of individuals.^7^

The levels of bone Gla-protein (BGP or osteocalcin) and bone alkaline phosphatase (B-ALP) reflect the changes of bone formation most sensitively and speciﬁcally, while that of C-terminal telopeptide of type I collagen (CTX) indicates the changes of bone resorption most satisfactorily.^8^ Earlier studies regarding bone metabolism, which relied primarily on urinary markers such as deoxypyridinoline and pyridinoline, were time-consuming and cumbersome, thus rendering the measurement imprecise. However, CTX, as a serum marker, is preferred for measuring bone resorption^9^ because it is highly correlated to bone turnover rate and available for detection in laboratory.^10^

Although the three biomarkers have been used to detect bone metabolism, they have seldom been utilized in clinical practice simultaneously. Thereby motivated, the expressions and clinical significance of BGP, B-ALP and CTX for OP patients were investigated in this study, aiming to provide evidence for developing individualized treatment plans.

## METHODS

**Fig.1:**
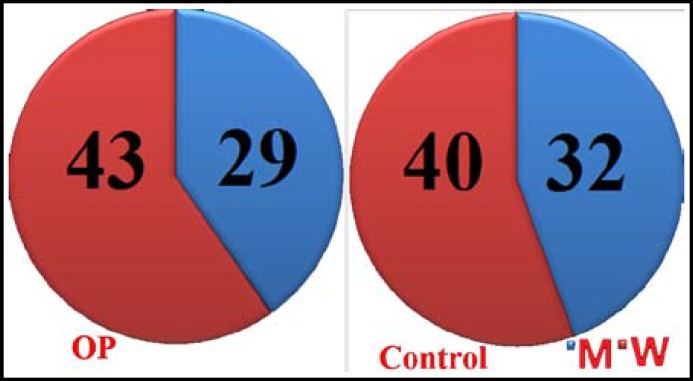
General information of OP patients and controls.

***General information: ***This study has been approved by the ethics committee of the 210th Hospital of People's Liberation Army. Written informed consent was obtained from all participants and all clinical investigations have been conducted according to the principles expressed in the Declaration of Helsinki. Seventy-two patients diagnosed as OP in our hospital from March 2011 to March 2013 were selected as the OP group, including 29 males and 43 females, aged 52-71 years old (average: 60.72 ± 9.58). Another 72 healthy volunteers were randomly selected as the control group, including 32 males and 40 females, aged 49-66 years old (average: 59.18 ± 9.31). The gender and age of the two groups did not differ significantly (P>0.05) (Fig.1).

***Evaluation criteria: ***Patients were diagnosed according to the criteria in "Chinese guideline on prevention and treatment of primary osteoporosis"^11^ issued by Chinese Medical Association of Osteoporosis and Mineral Disease. Meanwhile, the patients with secondary hyperparathyroidism and secondary OP induced by adrenocorticotropic hormone and diabetes, as well as those who took anti-OP agents recently were excluded.

***Methods:*** The BGP, B-ALP and CTX levels of the two groups were measured by collecting fasting venous bloods. BGP level was detected by radioimmunoassay (Shanghai Ricky Biotechnology Co., Ltd.), and B-ALP (Shanghai Jining Industry Co., Ltd.) and CTX levels (Sirocco Biotechnology (Shanghai) Co., Ltd.) were determined by enzyme-linked immunosorbent assay. BMD values were measured by XR-600 quick scanning machine (NORLAND, USA). All the measurements were performed strictly according to "National Guide to Clinical Laboratory Procedures"^12^ and instructions of apparatus and reagents.

***Observation indices: ***The correlations between BGP, B-ALP and CTX levels and BMD values were investigated and compared.

***Statistical analysis: ***All data were analyzed by SPSS 17.0. The measurement data were expressed as mean ± standard deviation ( ) and compared by t test. The correlations between BMD values and bone metabolism indices were subjected to linear correlation analysis. P<0.05 was considered statistically significant.

## RESULTS

***Comparison between bone metabolism indices: ***The BGP levels of the OP group [(5.61±5.52) ng/ml] was significantly higher than that of the control group [(3.24±1.28) ng/ml] (P<0.05), but the B-ALP and CTX levels of the two groups had no significant differences (P>0.05) (Table-I).

***Correlations between BMD values and bone metabolism indices of the OP group: ***The BMD values of femoral neck and Ward's triangle in the OP group were negatively correlated with the B-ALP levels (P<0.05). For women OP patients, the BMD values of femoral neck and Ward's triangle were also negatively correlated with the B-ALP levels (P<0.05). However, the BGP and CTX levels of OP patients were not significantly correlated with the BMD values. In the meantime, the B-ALP levels of male patients were also not significantly correlated with BMD (Table-II).

***Correlations between BMD values and bone metabolism indices of the control group: ***The BMD values of femoral neck were significantly negatively correlated with the CTX level (P<0.05), whereas it were not significantly correlated with the BGP and B-ALP levels. In contrast, the BMD values of greater trochanter and Ward's triangle were not significantly correlated with the BGP and B-ALP levels (Table-III).

## DISCUSSION

There is a dynamic equilibrium between human bone formation and resorption, which maintains stable bone mass and BMD. In case of hormone level changes and calcium deficiency, the equilibrium is destroyed, thus inducing OP by decreasing bone mass and BMD. OP, which is prevalent among the elderly and menopause women, is associated with enhanced bone resorption with/without deficient bone formation.^13^^,^^14^ With disease progression, OP patients are prone to bone fracture owing to remarkably reduced bone mineral content and intensity, the health and quality of life of whom are thus evidently affected.^15^^,^^16^ Hence, OP patients should be treated based on the pathogeneses of individuals.

OP occurs when bone resorption exceeds formation. BGP and B-ALP levels are related with bone formation, and CTX level is related with bone resorption.^17^ Clinically, the disease can be classified as primary type 1 OP (postmenopausal OP) and primary type 2 OP (senile osteoporosis). Type 1 OP is associated with postmenopausal endocrine changes, accompanied by raised levels of bone metabolism indices. Contrarily, type 2 OP, which results from ageing, is concomitant with normal or reduced levels of bone metabolism indices.^18^ Clarifying bone metabolism changes can guide clinical administration. Despite the ability to detect bone metabolism, biopsy, which is traumatic, cannot be widely applied in clinical practice.^19^ Therefore, we herein detected the variations of bone metabolism indices.

**Table-I:** Comparison between bone metabolism indices

**Group**	**Case number (n)**	**BGP (ng/ml)**	**B-ALP (ng/ml)**	**CTX (U/L)**
OP group	72	5.61±5.52^a^	0.43±0.37	31.98±12.64
Control group	72	3.24±1.28	0.37±0.42	30.71±7.53

Compared with the control group,

a *P*<0.05.

**Table-II:** Correlations between BMD values and bone metabolism indices of the OP group (r values).

**Position**	**OP group (n=72)**			**Male (n=29)**			**Female (n=43)**		
	**BGP**	**B-ALP**	**CTX**	**BGP**	**B-ALP**	**CTX**	**BGP**	**B-ALP**	**CTX**
Femoral neck	-0.047	-0.220^a^	0.089	0.162	0.318	0.031	-0.186	-0.316^a^	-0.091
Greater trochanter	0.033	-0.171	0.171	0.193	-0.158	0.018	-0.086	-0.056	-0.022
Ward's triangle	-0.094	-0.287^a^	-0.016	0.057	0.014	-0.194	-0.206	-0.366^a^	0.007

a *P*<0.05.

**Table-III:** Correlations between BMD values and bone metabolism indices of the control group (r values).

**Position**	**Control group (n=72)**		
	**BGP**	**B-ALP**	**CTX**
Femoral neck	0.005	-0.032	-0.436^a^
Greater trochanter	0.047	-0.195	0.208
Ward's triangle	-0.149	-0.055	-0.198

a *P*<0.05.

Although bone metabolism indices cannot diagnose OP, they are capable of evaluating bone turnover rate.^20^ In this study, only the BGP level of the OP group was significantly higher than that of the control group, indicating most enrolled patients were type 1 OP. In the OP group, the B-ALP levels were negatively correlated with the BMD values of femoral neck and Ward's triangle. Although OP women had identical outcomes, OP men did not have such correlations. The findings reveal that OP patients should be treated according to their own pathophysiological conditions, being consistent with a previous study.^21^ Furthermore, we herein combined three eligible biomarkers for detecting bone metabolism, thus allowing this study valuable.^22^^,^^23^

In summary, bone metabolism can be evaluated by detecting BGP, B-ALP and CTX levels, which inspires more thorough clinical typing and development of treatment protocols. Further studies on combining the three indices in OP detection are still ongoing.

## Authors contribution:

**XL: **Conceived, designed the experiments and prepared the manuscript.

**DZ, JW, YL** **: **Performed the experiments and analyzed the data.

## Notes

***Conflicts*** ***of interest*** ***: ***The authors declare no conflicts of interest.
